# Assessment of External Properties for Identifying Banana Fruit Maturity Stages Using Optical Imaging Techniques

**DOI:** 10.3390/s19132910

**Published:** 2019-07-01

**Authors:** Jiajun Zhuang, Chaojun Hou, Yu Tang, Yong He, Qiwei Guo, Aimin Miao, Zhenyu Zhong, Shaoming Luo

**Affiliations:** 1 Academy of Contemporary Agricultural Engineering Innovations, Zhongkai University of Agriculture and Engineering, Guangzhou 510225, China; 2 College of Biosystems Engineering and Food Science, Zhejiang University, Hangzhou 310058, China; 3 Guangdong Key Laboratory of Modern Control Technology, Guangdong Institute of Intelligent Manufacturing, Guangzhou 510070, China

**Keywords:** maturity stage, banana fruits, optical imaging technique, external properties, image processing

## Abstract

The maturity stage of bananas has a considerable influence on the fruit postharvest quality and the shelf life. In this study, an optical imaging based method was formulated to assess the importance of different external properties on the identification of four successive banana maturity stages. External optical properties, including the peel color and the local textural and local shape information, were extracted from the stalk, middle and tip of the bananas. Specifically, the peel color attributes were calculated from individual channels in the hue-saturation-value (HSV), the International Commission on Illumination (CIE) L*a*b* and the CIE L*ch color spaces; the textural information was encoded using a local binary pattern with uniform patterns (UP-LBP); and the local shape features were described by histogram of oriented gradients (HOG). Three classifiers based on the naïve Bayes (NB), linear discriminant analysis (LDA) and support vector machine (SVM) algorithms were adopted to evaluate the performance of identifying banana fruit maturity stages using the different optical appearance features. The experimental results demonstrate that overall identification accuracies of 99.2%, 100% and 99.2% were achieved using color appearance features with the NB, LDA and SVM classifiers, respectively; overall accuracies of 92.6%, 86.8% and 93.4% were obtained using local textural features for the three classifiers, respectively; and overall accuracies of only 84.3%, 83.5% and 82.6% were obtained using local shape features with the three classifiers, respectively. Compared to the complicated calculation of both the local textural and local shape properties, the simplicity and high accuracy of the peel color property make it more appropriate for identifying banana fruit maturity stages using optical imaging techniques.

## 1. Introduction

Due to their abundant nutritional elements, bananas play a key role in the human diet, and they are the fourth most important food crop worldwide [1]. China is one of the main banana-growing and producing countries. Since bananas are not usually allowed to ripen on the tree to reach peak maturity [2], the maturity stage of harvested bananas plays an important role in both the shelf life and market price. Ripening bananas in similar maturity stages can usually guarantee fruits with better external qualities within the same batch and can ultimately be conducive to prolonging the shelf life [3,4]. Therefore, it is important to identify and establish accurate banana maturity stages for postharvest consumption and marketing, a key aspect of which is to sort fruits with different maturity stages or qualities automatically [5,6]. An automatic fruit sorting system usually consists of conveyor belts, computer vision and control modules [7]. The first important processing step in such systems involves the computer vision (including RGB and other spectral imaging techniques) module, which is used for fruit identification; then, the classified fruits with different maturity stages or qualities will be delivered to corresponding banks by the conveyor belts and control module.

The identification of fruit maturity stages can be conducted using indices that are relevant to internal attributes such as titratable acidity (TA) [8,9,10], flesh firmness [11,12], soluble solids content (SSC) [9], starch content [13,14] and total soluble solids (TSS) [15]. However, precisely measuring the above indices usually requires manual destructive sampling inspection, which is costly, nontrivial and laborious and not appropriate for on-line estimation of fruit maturity [6] and is thus not suitable to be integrated in automatic fruit sorting systems. Therefore, most studies emphasize the development of alternative nondestructive, efficient methods by measuring internal or external attributes such as peel color and spectral reflectance that can be easily implemented in a computer vision module. Khodabakhshian et al. [16] determined four different maturity stages in pomegranates based on quality factors including firmness and TSS measured by a multispectral imaging device, and the resultant spectral information was modeled using a partial least squares regression algorithm with a correlation coefficient larger than 0.93. Li et al. [17] investigated the maturity classification of cherry fruits by the SSC and pH value measured using near-infrared hyperspectral imaging techniques; optimal feature bands were first selected from high-dimensional spectral data by a genetic algorithm, and a multiple linear regression model was built to estimate the maturity of cherry fruit that achieved a correct classification ratio of 96.4%. The spectral properties of bananas at wavelengths of 532, 660, 785, 830 and 1060 nm from different maturity stages were investigated by Adebayo et al. [18]; a positive correlation between the maturity stage and a reduced scattering coefficient was reported. Xie et al. [19] studied the feasibility of identifying banana fruit maturity using a hyperspectral imaging device at wavelengths ranging from 380 nm to 1023 nm and reported that partial least squares modeling accurately predicted mature and immature bananas using both the color and firmness properties extracted from hyperspectral data. Mohammadi et al. [20] graded persimmons into three maturity stages using image analytical techniques, where the external color information of persimmons was encoded in red-green-blue (RGB) color space and fed into a quadratic discriminant model, achieving an overall grading accuracy of 90.24%. Based on image processing techniques, three fresh tomato maturity levels (green, orange and red) were identified by Wan et al. [21], where the average blue-channel intensity and the hues of pixels in the maximum inscribed circle within each tomato’s surface were extracted and then classified with a backpropagation neural network; the average accuracy of the three maturity levels reached 99.31%. Tan et al. [22] proposed a stepwise, computer vision-based algorithm to recognize the maturity stages of blueberries (mature, intermediate and young), and the recognition pipeline attained an average accuracy of 92.07%; specifically, the fruit regions were first located using histogram-oriented gradients and feature attributes in the International Commission on Illumination (CIE) L*a*b* color space, and then the maturity of a located blueberry was determined using template matching with a weighted Euclidean distance. Marimuthu et al. [23] formulated a particle swarm optimized fuzzy model to grade banana fruits into unripe, ripe and overripe stages using peel color attributes extracted from the hue channel and opponent colors in CIE L*a*b* space, achieving an average classification accuracy of 93.11%.

In recent decades, much effort has been made in studying how to recognize fruit maturity stages, illustrating the remarkable potential of methodologies using hyperspectral and optical imaging techniques. Compared to the internal properties, external properties such as color and textural information provided by many fruits can usually be extracted much easier and serve as important indicators for evaluating ripeness [4,24]. However, most of the current work using external properties has focused on a single type of physical property (e.g., color) to grade the maturity stage, especially for the task of recognizing banana ripeness, yet it is also interesting to further understand under which circumstances each external property performs better. Hence, this study investigates the importance of identifying the maturity stage of banana fruits by different external properties, including color, local texture and local shape, using optical imaging techniques.

## 2. Materials and Methods

### 2.1. Data Acquisition

**Figure 1:**
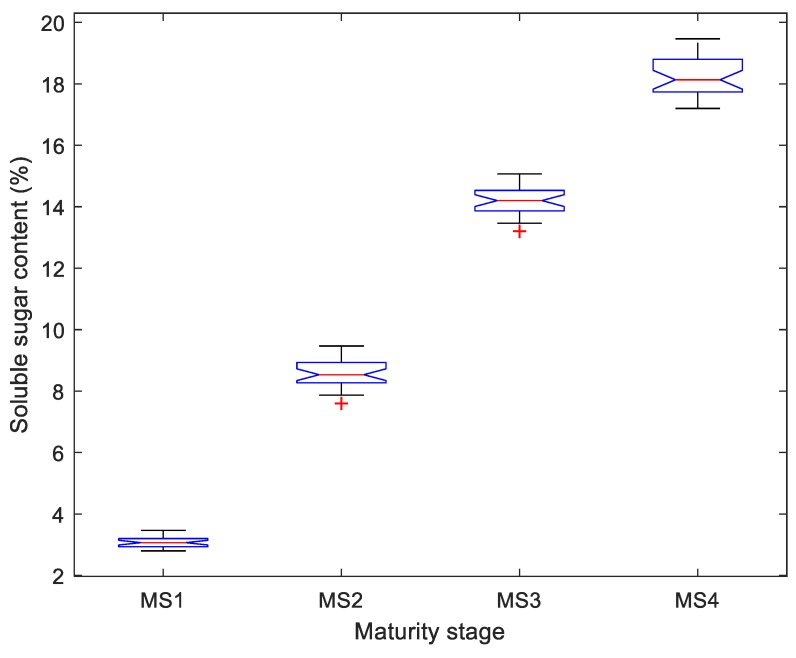
Distribution of soluble sugar content in bananas at different maturity stages.

**Figure 2:**
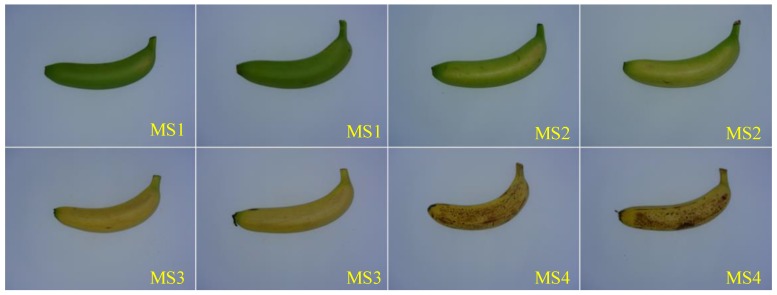
Some example images of bananas in the dataset.

Cavendish banana fruits at four successive maturity stages, i.e., stage 1 (MS1) indicating an all-green banana, stage 2 (MS2) indicating a mostly yellow banana with some or a little bit of green, stage 3 (MS3) indicating an all yellow banana and stage 4 (MS4) indicating a mostly yellow banana with some or many brown spots, were prepared from a local fruit market in Guangzhou, China. The purchased bananas were of similar sizes and shapes and were stored at 12–15 ℃ in a laboratory environment, and some of the bananas were disfigured by slight defects during transportation. In total, 441 banana samples (116, 109, 109 and 107 samples from the MS1, MS2, MS3, and MS4 maturity stages, respectively) were prepared. As a baseline, 120 bananas at different maturity stages (30 bananas per stage) were selected and the soluble sugar content of some of the 120 bananas was first destructively measured using a portable refractometer (Model BK-506, Shanghai Dingleng Industrial Co., Ltd., Shanghai, China). The resultant soluble sugar content is the average value from three positions (i.e., the stalk, middle and tip) on any banana fruit. Figure 1 illustrates the distribution of the soluble sugar content measured from bananas in the four maturity stages, indicating that there is probably a positive relationship between the visible, external properties and the soluble sugar content of bananas at various maturity stages. Then, the remaining 321 bananas (86, 79, 79 and 77 samples from the MS1, MS2, MS3, and MS4 maturity stages, respectively) were used for the imaging phase. Each banana was placed on the center of a plain, partial white cloth approximately 60 cm immediately under a charge-coupled device (CCD) camera (Model H1600Cam, Ruishi Instrument Equipment Co., Ltd., Shenzhen, China). In total, 200 images (50 bananas per stage) were randomly selected and served as the training dataset, and the remaining 121 images formed the test dataset. Some examples of the resultant images are shown in Figure 2.

### 2.2. Methods for Assessing Performance of Identifying Banana Maturity Stages Using External Properties

**Figure 3:**
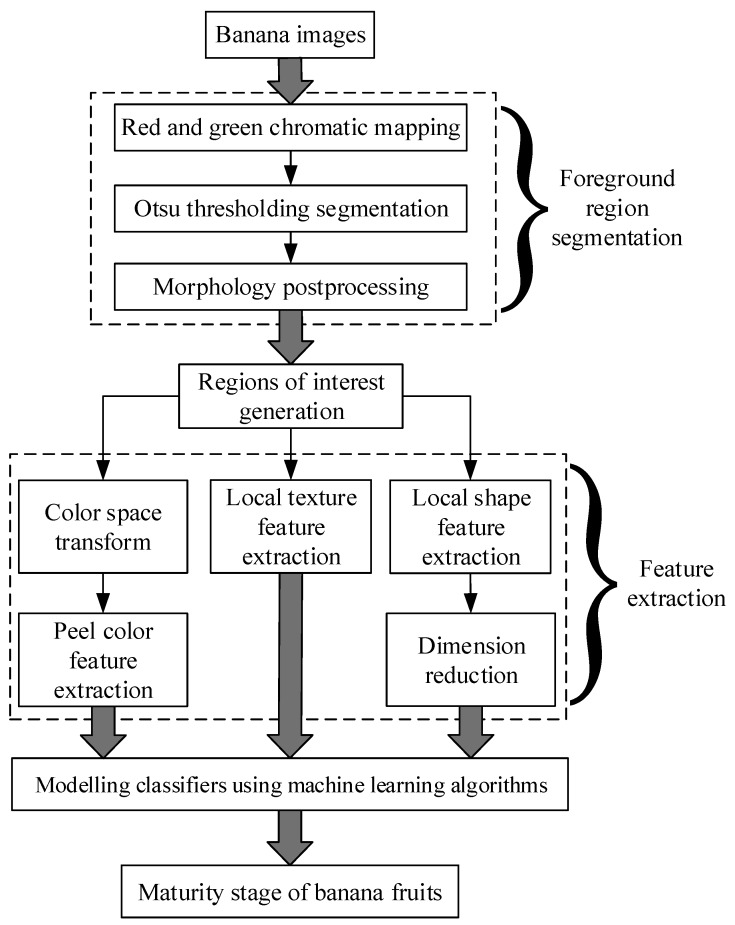
Flow diagram of the presented method.

The change in the maturity stage is associated with obvious external appearance variability in bananas, such as the peel color, and the local texture and shape, which are influenced by brown spots. Therefore, a combination of image feature extraction and pattern recognition techniques could be an alternative method for identifying the maturity stage of bananas. Figure 3 illustrates the main procedures for the identification of banana fruit maturity stages: foreground region segmentation, feature extraction, and maturity stage classification.

#### 2.2.1. Foreground Region Segmentation

**Figure 4:**
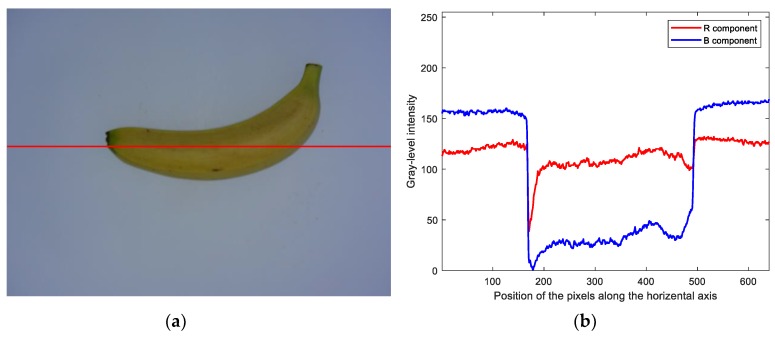
Comparison of the color intensity between the banana and background regions: (**a**) a red horizontal scan line across the banana fruit region, and (**b**) the distribution of gray-level intensities of pixels from both the banana and background regions specified by the horizontal scan line.

For any horizontal scan line across banana fruit, as shown in Figure 4a, the color intensity of pixels of the banana is different from that of the background. Specifically, in RGB color space, the gray-level intensity of pixels within the banana region in the red (*R*) component is always higher than that in the blue (*B*) component, as shown in Figure 4b, while the gray-level intensity of pixels within the background region in the *R* component is always lower than that in the *B* component. Therefore, the banana could be separated from the background using the following red and blue (RB) chromatic mapping:

(1) $$I_{RB}=R-B$$

where *I_RB_* is the calculated RB chromatic map, and *R* and *B* refer to the red and blue components of the input image in RGB color space, respectively.

Since the hue and appearance of the banana images in the dataset could differ to some extent due to slight changes in lighting conditions during imaging, it is hard to always ensure consistently high intensities of all the pixels within the banana, and thus, the resultant RB chromatic map is not a binary image that separates the banana fruit from the background. Therefore, the Otsu thresholding algorithm [25] was adopted to segment the foreground banana region from *I_RB_*. Then, the mathematical morphology open operation followed by the hole-filling operation was used to filter out noise and fill holes in the binary image obtained by the Otsu thresholding algorithm, respectively, and the foreground banana region was extracted from the morphologically postprocessed binary image. 

**Figure 5:**
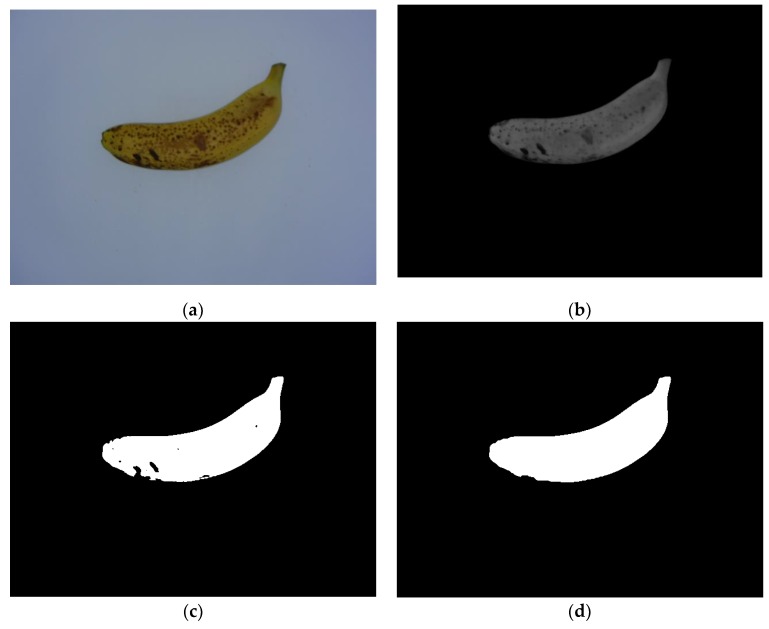
Foreground region segmentation: (**a**) the input RGB image, (**b**) the resultant RB chromatic map of (**a**), (**c**) the binary segmentation of (**b**) using the Otsu thresholding algorithm, and (**d**) the postprocessed binary image of (**c**) using mathematical morphology operations.

Figure 5 illustrates an example of banana fruit region segmentation using a sample image from MS4. Figure 5b shows that most of the background was filtered out in the resultant RB chromatic map because the difference in intensity between the *R* and *B* components of the background is much lower than that from the banana fruit region. Moreover, a large number of brown spots were distributed on the surface of the banana peel, and thus, no significant RB intensity difference between the brown spots and the background was found, which resulted in an incorrect segmentation of some brown spots, as shown in Figure 5c. Fortunately, mathematical morphology image processing techniques, i.e., open operations (using the disk-shaped structural element with a 10-pixel radius) followed by hole filling, could fix the incorrectly segmented foreground regions, as shown in Figure 5d, and more accurate banana fruit regions could be extracted for the following procedures.

#### 2.2.2. Feature Extraction

The external optical properties of bananas, including the color, local texture and shape features that were observed from samples belonging to various maturity stages in the training dataset, are potential criteria for identifying different maturity stages. For example, the local texture and shape structure are significantly affected by the distribution of brown spots on the peel at higher banana maturity stages.

**Figure 6:**
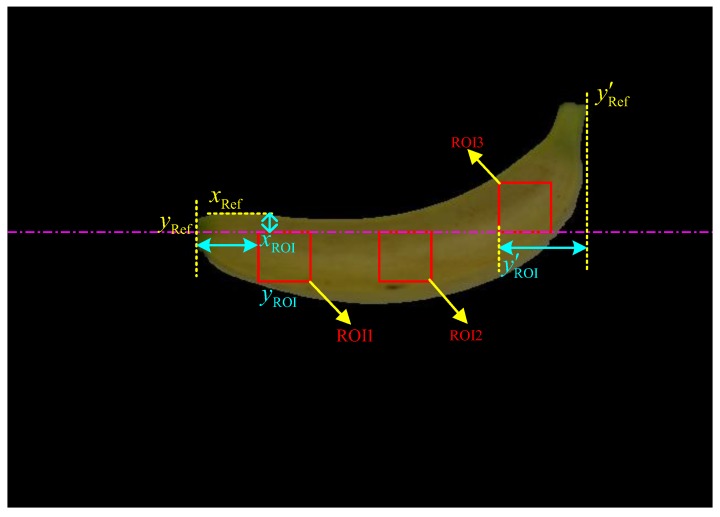
Location of three ROIs from the segmented banana region: the size of each ROI is 48 pixels × 48 pixels.

First, as illustrated in Figure 6, three regions of interest (ROIs) were sampled and extracted from the segmented foreground banana region, where $x_{\mathrm{Ref}}$ is the upper-left horizontal boundary of the segmented banana, $y_{\mathrm{Ref}}$ and $y_{\mathrm{Ref}}^{{}^{\prime }}$ represent the left and right vertical boundaries of the segmented banana, respectively, $x_{\mathrm{ROI}}$ is the abscissa of the image origin of both ROI1 and ROI2, $y_{\mathrm{ROI}}$ and $y_{\mathrm{ROI}}^{{}^{\prime }}$ refer to the ordinate of the image origin of ROI1 and ROI3, respectively. The sizes of each ROI were assigned as 48 pixels × 48 pixels to simplify the following feature extraction process. By introducing different offsets, the value of $x_{\mathrm{ROI}}$ was determined by the value of $x_{\mathrm{ROI}}=x_{\mathrm{Ref}}+a$, where *a* is an integer ranging from 15 to 35 pixels, the value of $xy_{\mathrm{ROI}}$ was determined from $y_{\mathrm{ROI}}=y_{\mathrm{Ref}}+\left(y_{\mathrm{Ref}}^{{}^{\prime }}+y_{\mathrm{Ref}}\right)*\alpha$, where *α* is a random floating value ranging from 0.1 to 0.2, and the value of $y_{\mathrm{ROI}}^{{}^{\prime }}$ was determined from $y_{\mathrm{ROI}}^{{}^{\prime }}=y_{\mathrm{Ref}}^{{}^{\prime }}-\left(y_{\mathrm{Ref}}^{{}^{\prime }}+y_{\mathrm{Ref}}\right)*\beta$, where *β* is a floating value ranging from 0.15 to 0.25. Furthermore, the ordinate of the image origin of ROI2 was calculated by $\frac{y_{\mathrm{ROI}}+y_{\mathrm{ROI}}^{{}^{\prime }}}{2}$, and the abscissa of the image origin of ROI3 was determined by the value of $x_{\mathrm{ROI}}\;$– 48. Similar to the sampling method adopted in most destructive analysis methods, the sampling of three ROIs from several nonintersecting parts (i.e., the stalk, middle and tip positions) could improve the sequent identification performance.

(1) Color Feature Extraction

As the banana maturity changes from MS1 to MS4, the hue color gradually changes from full green to yellow, which is an important visual clue to discriminate various maturity stages. However, all the red, green and blue components in RGB color space contain both the color and brightness information of objects at the same time, where the brightness information might not be helpful for identifying the maturity stage. Therefore, to isolate only the color component, the hue-saturation-value (HSV), CIE L*a*b* and CIE L*ch color spaces were introduced to provide the pure color information because the color components in these spaces are independent of the corresponding brightness component.

For the three ROIs located at any segmented foreground banana region, the corresponding ROI images were first transformed from RGB color space into HSV, CIE L*a*b* and CIE L*ch color spaces. The hue component *H* and the saturation component *S* of the HSV color space, the a* color component and the b* color component of the CIE L*a*b* color space, and the chromatic degree component *c* and the hue component *h* of the CIE L*ch color space were extracted. Thus, the average intensity of all the pixels in each color component was calculated and served as a corresponding color feature value. All the feature values obtained from different color components (i.e., *H*, *S*, a*, b*, *c* and *h*) formed a 6-dimension color descriptor. Since there were three ROIs in each banana sample, the three color descriptors generated from different ROIs were then successively concatenated into a color feature vector with a dimension of 6 × 3 = 18.

(2) Local Texture Feature Extraction

The increase in the maturity could alter the local texture of a banana peel, which might be attributed to (i) a slight change in local gray-level intensity discontinuity caused by the color change across adjacent maturity stages or (ii) a gradual increase in brown spots while banana fruits reach close to the overmature stage. Therefore, textural information described by the local binary pattern (LBP) algorithm [26] was adopted to identify the maturity stage of banana fruits.

**Figure 7:**
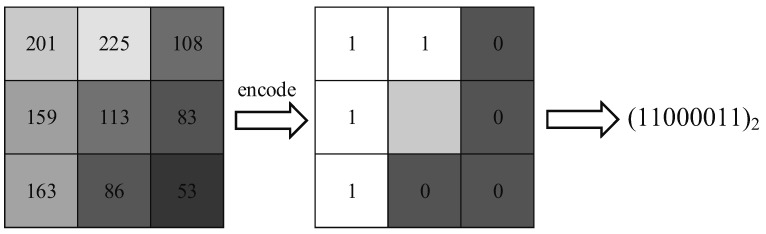
An example of the feature encoding procedure using an LBP descriptor.

LBP encodes any local 3 × 3 image region into a specific binary pattern, as shown in Figure 7. Basically, LBP compares the intensity of a center pixel *i_c_* and that of the 8 surrounded neighboring pixels *i_k_* (*k* = 1, 2,…, 8). The *k*-th neighboring pixel would be given a value of 1 if $i_{k}\geq i_{c}$ is true; otherwise, it would be given a value of 0. All the encoded values form an 8-bit binary pattern to depict the local intensity continuity in terms of equation (2). Therefore, a 256-dimension texture feature vector could be generated. However, it is not advisable to extract 256-dimensional LBP features from small ROIs; the result would be too sparse. Therefore, an LBP with a uniform pattern (UP-LBP) [27] was adopted to reduce the dimension of the resultant texture feature vector, where at most two conversions between adjacent encoded values (e.g., converting from a value of 1 to a value of 0 or vice versa) in an 8-bit binary number were considered. In total, 58 binary patterns meet the conversion rules, and the remaining 198 patterns are considered the 59th pattern. A 59-dimensional texture descriptor could be extracted from each ROI, and the resulting three texture descriptors generated from different ROIs were then successively concatenated into an UP-LBP texture feature vector with a dimension of 59 × 3 = 177.

(2) $$f_{LBP-8}=\sum_{k=1}^{8}2^{k-1}\times sign\left(i_{k}-i_{c}\right)$$

(3) Local Shape Feature Extraction

Similar to the change in local textures, the local shapes of the banana peel could also be influenced by the increase in the maturity stage. For example, the change in the gray-level intensity discontinuity across adjacent maturity stages would influence the distribution of local intensity gradients. Therefore, histogram of oriented gradients (HOG) [28] was adopted. The main idea of HOG is that local shape information can be well described by calculating the distribution of local edge directions and intensity gradients on a dense grid.

When extracting the HOG features, each ROI was equally divided into 4 × 4 image cells of 12 pixels × 12 pixels. Adjacent 2 × 2 cells formed an image block, and thus 3 × 3 blocks were generated. The size of the intersection area between two adjacent blocks was assigned by 1 cell × 2 cells in the horizontal dense scan or by 2 cells × 1 cell in the vertical dense scan. The gradients of the pixels within each cell were calculated using the Prewitt operator, and then the magnitude of the gradients was cumulatively voted into 9 uniformly spaced bins ranging from 0 to *π* according to the gradients’ direction. Thus, a 36-dimensional histogram was generated for each block and further normalized by the L1-norm. Therefore, a shape descriptor with a dimension of 36 × 3 × 3 = 324 could be extracted from each ROI, and the three shape descriptors generated from different ROIs were then successively concatenated into a HOG feature vector with a dimension of 324 × 3 = 972.

#### 2.2.3. Classification of the Maturity Stages

In the maturity stage identification task, it is interesting to evaluate the benefits from different types of external optical properties. The naïve Bayes (NB), linear discriminant analysis (LDA) and support vector machine (SVM) classifiers were used to model the extracted color, the local texture and the local shape features, respectively.

(1) NB Classifier

Let *m* labeled samples (extracted feature vectors) from *c* different classes in the training dataset be denoted by $X={\left\{\left(x_{i},y_{i}\right)\right\}}_{i=1}^{m}$, where $x=\left(x_{1},x_{2},\ldots ,x_{d}\right)$ and $y_{i}\in \left\{1,2,\ldots ,c\right\}$, and a test sample be denoted by $x^{\prime }=\left(x_{1}^{\prime },x_{2}^{\prime },\ldots ,x_{d}^{\prime }\right)$. The NB classifier [29] is a supervised machine learning algorithm that is derived from Bayes’ theorem. The label of the *d*-dimensional feature vector $x^{\prime }$ can be predicted as follows:

(3) $$h_{\mathrm{NB}}\left(x^{\prime }\right)=\underset{c\in Y}{\arg\max}P\left(c\right)\prod_{i=1}^{d}P\left(x_{i}^{\prime }|c\right)$$

where *P*(*c*) is the prior probability of each class and $P\left(x_{i}^{\prime }|c\right)$ refers to the conditional probability of the feature $x_{i}^{\prime }$. Both *P*(*c*) and $P\left(x_{i}^{\prime }|c\right)$ can be estimated from the training dataset.

(2) LDA Classifier

LDA is a variant of Fisher’s discriminant analysis [30] and is suitable for multiclass classifications; the basic idea is to minimize the within-class variance and maximize the between-class variance in the training samples. Suppose that the average feature vector for the *i*-th class $X_{i}$ is $\mu_{i}$ and the population average feature vector for all the *c* classes is ***μ***. LDA aims to find the optimal classification hyperplane from the projected feature space indicated by:

(4) $$\underset{w}{\max}\frac{tr\left(w^{T}S_{b}w\right)}{tr\left(w^{T}S_{w}w\right)}$$

where w is the projecting matrix and ***S**_w_* and $S_{b}$ refer to the within-class and between-class scatter matrix, respectively, which is denoted as follows:

(5) $$S_{w}=\sum_{i=1}^{c}\sum_{x\in X_{i}}\left(x-\mu_{i}\right){\left(x-\mu_{i}\right)}^{T}$$

(6) $$S_{b}=\sum_{x\in X}\left(x_{i}-\mu \right){\left(x_{i}-\mu \right)}^{T}-S_{w}$$

Once the test feature vector $x^{\prime }$ is projected using w, the label of $x^{\prime }$ will be assigned to that of the class whose cluster center is closest to the projected $x^{\prime }$. Note that the small sample size problem [31] would occur if the number of training samples is less than the dimension of the feature vectors, and thus, the objective function indicated by Equation (4) cannot be directly solved. To avoid this problem, when higher-dimensional feature vectors are provided, principal component analysis (PCA) is first adopted to reduce the feature dimension, and then the resultant lower-dimensional feature vectors will be fed to the LDA for classification.

(3) SVM Classifier

The SVM algorithm was first introduced for binary classification task based on structural risk minimization rules [32]. Suppose that the training dataset $X={\left\{\left(x_{i},y_{i}\right)\right\}}_{i=1}^{m}$ is restricted by the condition $y_{i}\in \left\{-1,+1\right\}$, the SVM aims to find the optimal classification hyperplane by solving the following objection function:

(7) $$\begin{array}{l} F_{\mathrm{SVM}}\left(\omega ,\xi \right)=\frac{1}{2}{\left\|\omega \right\|}^{2}+C\sum_{i=1}^{m}\xi_{i}\\ s.t.y_{i}\left(\omega^{T}x_{i}+b\right)\geq 1-\xi_{i},\;C\geq 0,\;\xi_{i}\geq 0,\;i=1,2,\ldots ,m \end{array}$$

The solution of Equation (7) can be obtained by maximizing its dual form as follows:

(8) $$\begin{array}{l} \underset{\alpha }{\max}\sum_{i=1}^{m}\alpha_{i}-\frac{1}{2}\sum_{i=1}^{m}\sum_{j=1}^{m}\alpha_{i}\alpha_{j}y_{i}y_{j}K\left(x_{i},x_{j}\right)\\ s.t.\sum_{i=1}^{m}\alpha_{i}y_{i}=0,\alpha_{i}\geq 0,\;i=1,2,\ldots ,m \end{array}$$

where *K*(·) is a kernel function. To guarantee a fair comparison between the SVM and LDA classifiers, a linear kernel function was adopted, i.e., $K\left(x_{i},x_{j}\right)=x_{i}^{T}x_{j}$. The label of the test feature vector $x^{\prime }$ is predicted by the following decision function:

(9) $$h_{\mathrm{SVM}}\left(x^{\prime }\right)=\sum_{i=1}^{v}\alpha_{i}y_{i}K\left(x^{\prime },x_{i}\right)+b$$

To extend the SVM to multiclass classification task, the one-against-one technique was adopted to model a multiclass SVM classifier.

#### 2.2.4. Evaluation Metrics

The maturity stage identification performance from different combinations of external optical properties and classification algorithms was evaluated using the recall rate (*RR*) and the overall accuracy (*OA*) metrics, which are defined by:

(10) $$RR_{\mathrm{MS}i}=\frac{n_{\mathrm{MS}i}}{N_{\mathrm{MS}i}}\times 100\%$$

(11) $$OA=\frac{\sum n_{\mathrm{MS}i}}{\sum N_{\mathrm{MS}i}}\times 100\%$$

respectively, where *N*_MSi_ is the total number of samples in the *i*-th maturity stage in the test dataset and *n*_M*Si*_ is the number of correctly classified samples from the *i*-th maturity stage. The *RR* is a local evaluation metric indicating how accurately a classifier predicts each class of samples, while the *OA* is a global evaluation metric that measures the ratio of the total number of correctly classified samples in the whole test dataset to *N*_MSi_. A combination (e.g., color feature + SVM) of different features and classifiers is regarded as performing better in identifying banana maturity stages when higher values of both its *RR* and *OA* are achieved. Additionally, 10-fold cross-validation was first adopted using the training dataset to evaluate the training performance on different combinations of features and classifiers, which can help to observe the overfitting phenomenon [33]. Specifically, all the training samples would be randomly divided into ten disjoint sub-sets, where nine of the sub-sets were used to train different validation models and the remaining one was served as the validation data; note that, each model referred to a combination of one type of feature extraction algorithm and one type of classification algorithm, e.g., color feature + SVM, local texture feature + SVM, local shape feature + SVM, etc., and there were nine different models in total. To reduce the randomness of the samples partitioned in one single cross-validation procedure, the aforementioned 10-fold cross-validation was run 20 times independently for each validation model, in which the average recall rate of the 20 independent runs as well as the corresponding standard deviation were calculated. Furthermore, all the samples in the training dataset were used to train nine different identification models (e.g., color feature + NB, etc.), whose performance of identifying banana maturity stages would be evaluated using the test dataset.

## 3. Results and Discussion

**Figure 8:**
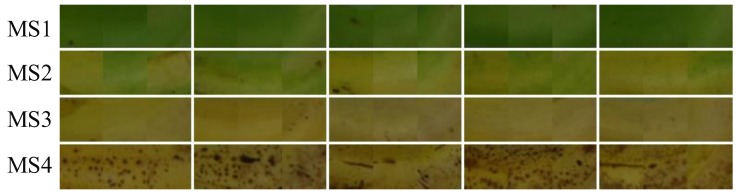
Examples of some 48 pixels × 48 pixels × 3-sized ICOs: each row of ICOs indicate different maturity stages.

To train the corresponding maturity stage classifiers with the NB, LDA and SVM algorithms, 200 image cutouts (ICOs) were generated from the training dataset and served as the training samples to model the classifiers. Fifty ICOs were from each maturity stage, and each ICO contained 3 different ROIs, as mentioned in Section 2.2.2. Some examples of the ICOs are shown in Figure 8. The performances of the methods were evaluated using the test dataset. All the experiments were performed using MathWorks MATLAB R2018a on a personal computer equipped with an Intel Core i5-8500 CPU and 16 GB of RAM.

The extracted color, UP-LBP and HOG features of the training samples (i.e., the 200 ICOs) were first fed to NB, LDA and SVM classifiers, respectively. Then, 20 independent iterations of 10-fold cross-validation were conducted for each combination of the extracted features and selected classifiers. The resultant average recall rate and standard deviation using different combinations are shown in Table 1. Compared with the following results of corresponding combinations using the test dataset as listed in Table 2, Table 3, Table 4, Table 5, Table 6, Table 7, Table 8, Table 9 and Table 10, these results demonstrate that consistent identification performance was achieved from both the training and test dataset, the samples from which were disjoint from each other.

### 3.1. Assessment of Using the Color Features to Identify Banana Maturity Stages

**Figure 9:**
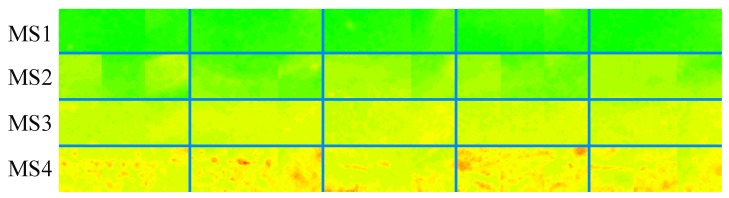
Color distributions of the ICOs shown in Figure 8.

The three extracted ROIs in each ICO were then transformed into the HSV, CIE L*a*b* and CIE L*ch color spaces. The average values of the *H*, *S*, a*, b*, *c* and *h* components within each ROI were calculated and concatenated to form a color descriptor, and the resultant 3 color descriptors were further concatenated to generate a whole color feature vector for the single sample image. The distribution of the hues of the ICOs shown in Figure 8 is illustrated in Figure 9, since hue information can be easily distinguished by human vision. Intuitively, according to Figure 9, significant differences could be found among the color distributions in the four maturity stages indicating that identifying the maturity stage of fruits using the color of banana peels is feasible and reliable.

**Figure 10:**
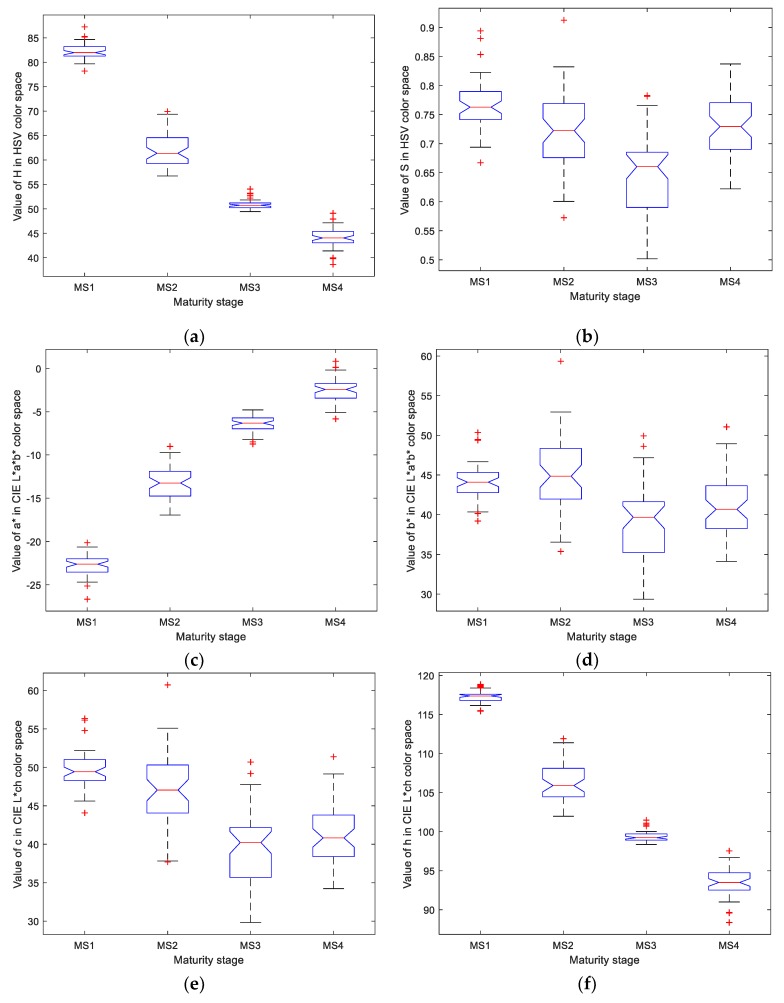
Distribution of the different color attributes from the training dataset: (**a**) the box plot of the *H* component, (**b**) the box plot of the *S* component, (**c**) the box plot of the a* component, (**d**) the box plot of the b* component, (**e**) the box plot of the *c* component, and (**f**) the box plot of the *h* component.

To further analyze how each color contributes to the identification of the maturity stages, Figure 10 gives the statistical distribution of different colors calculated from the training dataset. The results demonstrate that a significant difference was obtained among the four maturity stages based on the features extracted using the *H*, a* and *h* components, indicating that the maturity stage is directly related to the color of the banana peel, which and is in accordance with the findings reported in [4,6], where the measured chlorophyll content gradually degrades from the stage when the banana peel is green to that when the peel is corrupted with brown spots. On the other hand, the color attribute using other components could not provide more discriminative information among the four classes. For example, the saturation and the chromatic degree only describe the purity of a specific color, indicating the green hue of a banana peel at MS1 could represent a similar purity as a banana peel at MS3. Therefore, the color attribute provided by these components might not improve the maturity stage identification; thus, only the color attributes obtained from *H*, a* and *h* components should be considered to guarantee a higher prediction accuracy.

The extracted color feature vectors from the training dataset were used to train the NB, LDA and SVM classifiers. Based on the foreground region segmentation and the three color appearance-based classifiers, Table 2, Table 3 and Table 4 give the respective identification results on the test dataset.

**Figure 11:**
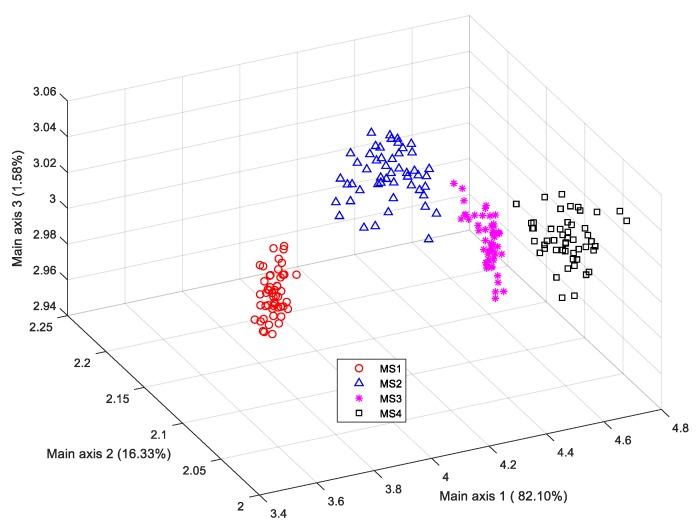
LDA scatter plot of samples using color features.

According to Table 2, Table 3 and Table 4, compared with the other classifiers, the color appearance-based LDA classifier achieved the best identification performance on the test dataset. Figure 11 illustrates the distribution of the four stages of training samples in the lower-dimension color feature space projected by the LDA classifier, where the value in parentheses along each main axis refers to the projection variance. 

This figure demonstrates that the first three projection directions (with 98.43% of the total accumulative projection variance) incorporate the most important information for the classification of the four banana maturity stages, since the scatter plot of the different groups achieved remarkable discriminability.

On the other hand, a high *OA* (99.2%) was attained with the SVM classifier. Specifically, an *RR* of 100% was attained in three classes (MS1, MS3 and MS4), while only 1 test sample from MS2 was incorrectly predicted as MS3, making the *RR* on MS2 reach 96.6%. Although the peel color of samples in MS2 is usually yellow with some green, while that in MS3 represents all yellow, the distribution of yellow hue on the banana peel might be random. Therefore, few resultant ROIs from MS2 might be located within yellow peel regions and thus, these singular samples might be incorrectly predicted as MS3 when using the color properties. Similar results were also found by Hou et al. [3]. Similarly, a good identification performance was also obtained with NB classifiers. The above results demonstrate that the color is suitable for correctly and reliably identifying different banana maturity stages.

### 3.2. Assessment of Using the Local Shape Features to Identify Banana Maturity Stages

**Figure 12:**
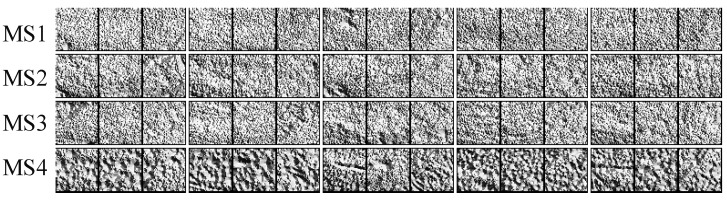
Visualization of the LBP texture features of ICOs from Figure 8.

Figure 12 shows the extracted LBP textures of the ICOs from Figure 8. Obviously, due to the distribution of brown spots on the banana peel, the local texture of the samples from MS4 is remarkably different from the other three maturity stages. However, it is difficult to distinguish the samples from MS1 to MS3 intuitively, and thus the identification using the UP-LBP texture feature was conducted by feeding these features to different classifiers. Table 5, Table 6 and Table 7 give the identification results on the test dataset using the NB, LDA and SVM classifiers, respectively. Note that since the dimension of the extracted UP-LBP texture feature was 177 and was smaller than the number of training samples (50 × 4 = 200), it was unnecessary to reduce the dimensions with PCA before training the LDA classifier.

**Figure 13:**
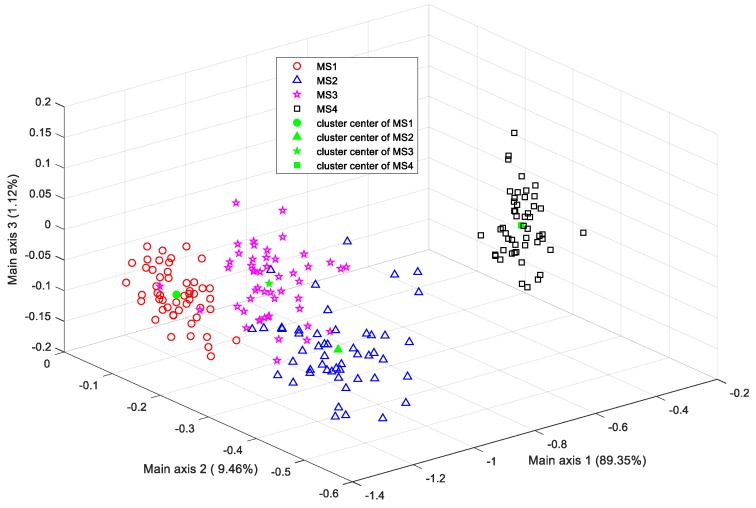
LDA scatter plot of the samples using local texture features.

The results shown in Table 5, Table 6 and Table 7 indicate that a better local prediction performance can be obtained on the test samples from MS4, where the *RR* reached 100% for each of the three different classifiers. Hence, local texture features are more suitable for recognizing overmature bananas. It could also be intuitively seen in the LDA scatter plot of the lower-dimension local texture feature space from the training samples (Figure 13), where the MS4 samples are farther away from the other maturity categories.

Compared to the results obtained using the color feature, more samples from MS1, MS2 and MS3 were misclassified using the UP-LBP feature, e.g., RRs of 97.2%, 93.1% and 82.8% were obtained by the SVM classifier for the test samples from MS1, MS2 and MS3, respectively. Ideally, more similar texture information would be provided by the samples from MS1 and MS3 due to their similar intensity distribution, compared to those from MS4; however, the increase in the ripening level might cause a slight intensity discontinuity in local regions, which would further change the local textures, especially for those banana peels with defects. Hence, most of the samples from MS1 and MS3 were correctly identified using the UP-LBP-based classifiers, except for some isolated samples. Moreover, some of the selected ROIs might be located near the yellow hue regions from the MS2 samples, which could result in similar local intensity discontinuities within the MS3 samples, which usually have yellow peels; hence, more samples from MS2 and MS3 were misclassified as each other.

The LDA classifier attained a worse global identification performance (with an *OA* of only 86.8%) than the SVM classifier, which is probably because the LDA-based prediction was simply determined by the minimum Euclidean distance between the test sample and the cluster center of each class, while the SVM classifier used an optimal decision hyperplane determined by the support vectors away from the classification margin. Therefore, unless descriptions with significant differences (e.g., the color feature) were provided, the LDA classifier might perform worse than the SVM classifier. Moreover, the different uniform patterns were generated based on the intensity discontinuity within nonoverlapping local windows, which probably guarantees independence among the attributes in the resultant UP-LBP feature vector. Hence, the NB classifier also achieved an acceptable global identification performance with an *OA* of 92.6%.

### 3.3. Assessment of Using the Local Shape Features to Identify Banana Maturity Stages

Due to the distribution of brown spots on the banana peel, remarkable differences in the local shape could be generated between the samples in the overmature stage and the other three classes. Therefore, MS4 bananas could be easily and accurately recognized by the different classifiers (i.e., the NB, LDA and SVM classifiers), as shown in the confusion matrices listed in Table 8, Table 9 and Table 10, respectively. The *RR* of the MS4 test samples reached 100% for all three different classifiers. However, some samples in the other maturity stages were incorrectly identified as MS4 when using the NB and LDA classifiers, which is likely because not all the samples were defect free. Even a small defect on an individual banana peel could change the local distribution of gradient orientations, making the resultant local HOG descriptor similar to some MS4 samples.

**Figure 14:**
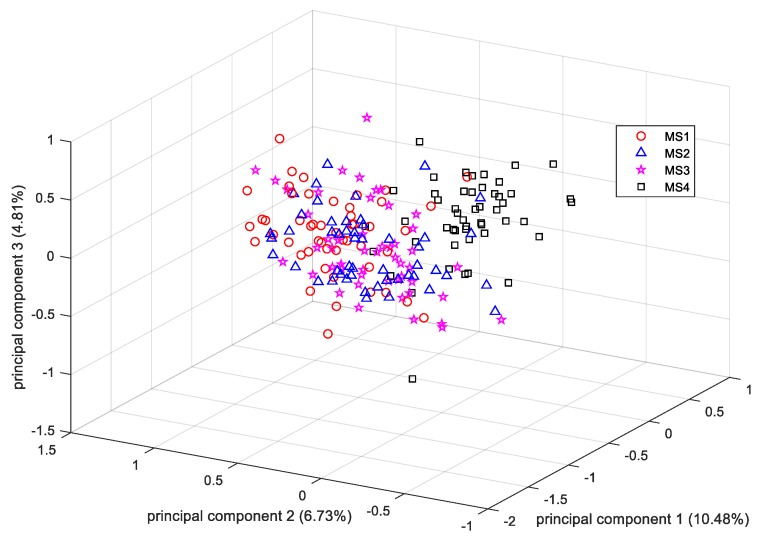
PCA scatter plot of samples using the HOG features.

**Figure 15:**
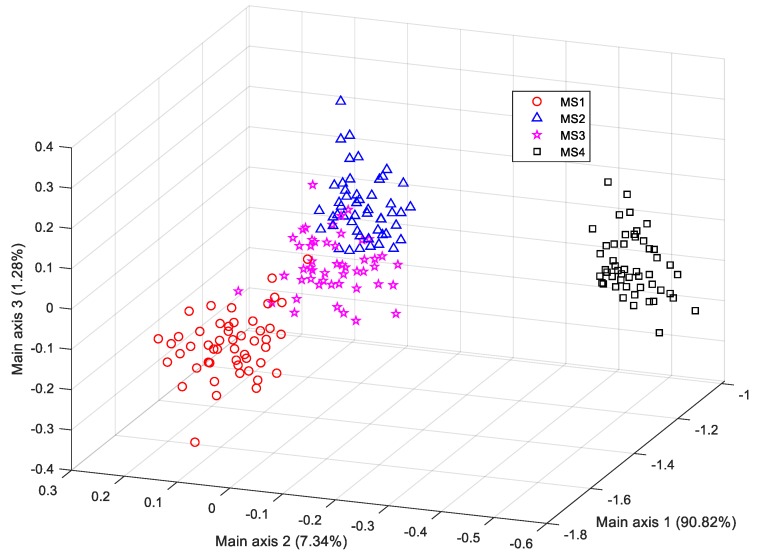
LDA scatter plot of samples using the HOG features after performing PCA.

Since the dimension of the resultant HOG feature was much larger than the number of training samples in our settings, PCA was adopted for dimension reduction of the HOG feature vectors before feeding them to the LDA classifier. Figure 14 gives the scatter plot of samples in the lower feature space projected by the PCA. The distribution demonstrates that there is no informative partition of the samples from the four maturity stages (the cumulative data variance in the first three components was only 22.02%). Probably because the PCA explains the samples distribution along the directions of higher variability in less amount of components and the wider the scatters distribute along a direction, the larger the corresponding samples variance is; however, the information generated by PCA might not always guarantee the ability to identify all the samples from different maturity stages [34], resulting in poor clustering results as shown in Figure 14. And according to [28], most of the generated histograms of oriented gradients along each orientation bin (i.e., the feature value of each attribute) provide equal importance for describing objects’ local shape information, indicating that the extracted HOG features might share similar or at least non-prominent data variance along the corresponding directions; thus, the transformed data in the feature space projected by PCA might not guarantee significant data variance along the first several directions, resulting in only approximately 20% of accumulative variance by the first three principal components. Therefore, more principal components should be considered to guarantee an acceptable identification performance using the dimensionally reduced HOG features. The first 100 components with a cumulative data variance of 90% were extracted and used to train the LDA classifier. Compared to the PCA, LDA aims to find the optimal projection directions in which the labelled samples from different maturity stages can be identified accurately, and thus it achieves larger variance in the main projection directions if the samples from different stages provide larger value of ratio of the between-class scatter and the within-class scatter [35]. Therefore, a more significant partition of the four different clusters was obtained in the resultant lower feature space projected by the LDA, as shown in Figure 15.

Compared with the *OA* obtained using the local texture-based feature, a poorer performance for identifying maturity stages using local shape features was found. The overall identification accuracies of 84.3%, 83.5% and 82.6% were obtained using HOG feature-based NB, LDA and SVM classifiers, respectively, according to the classification results illustrated in Table 8, Table 9 and Table 10. The HOG descriptor is different from the LBP descriptor, which globally encodes the local texture attributes by counting the number of different uniform patterns, in that the HOG descriptor simply concatenates all the local histograms of oriented gradients calculated from each block; thus, a slight defect on a banana peel might cause only a small change in specific local texture attributes. However, slight defects or intensity discontinuities would cause greater changes in the resultant HOG features. Therefore, the local texture features encoded by the LBP descriptor is more suitable for identifying the banana maturity stages than the local shape features encoded by the HOG descriptor.

## 4. Conclusions

This study investigated the importance of different external optical properties for identifying four successive banana fruit maturity stages. The main conclusions are as follows:

(1) Significant differences among the four maturity stages could be found in the *H* component in HSV color space, the a* component in CIE L*a*b* color space and the *h* component in CIE L*ch color space, resulting in an excellent *OA* of 99.2%, 100% and 99.2% using NB, LDA and SVM classifiers, respectively.
(2) With the combination of NB, LDA and SVM classifiers, a more acceptable overall identification accuracy of 92.6%, 86.8% and 93.4% was obtained using local texture features compared to 84.3%, 83.5% and 82.6% using local shape features, respectively, probably because the shape information encoded by the HOG descriptor is more sensitive to slight defects or changes in intensity discontinuities on banana peels than the textural information encoded by the UP-LBP descriptor.
(3) An *RR* of 100% for the MS4 bananas can be obtained using either the local texture or local shape features, due to the specific visual distributions of both the UP-LBP and HOG features generated by the brown spots. For the MS1 to the MS3 bananas, more samples were incorrectly identified using these two types of features, resulting in a worse local identification accuracy than the color appearance features, especially for the local shape features encoded by the HOG descriptor.
(4) The best identification performance could be achieved using the color feature-based classifiers, probably because the changes in banana maturity stages involve a more significant feature difference in appearance compared to those provided by both local texture and local shape appearance, which are more sensitive to the changes in local appearance (e.g., peel defects). The low-cost and easy-to-implement method using external optical properties makes it attractive for automatic banana sorting systems.

## Figures and Tables

**Table 1:** Cross validation results for each combination of features and classifiers.

	NB	LDA	SVM
Color	0.96 ± 0.03	0.99 ± 0.01	0.94 ± 0.04
UP-LBP	0.91 ± 0.06	0.88 ± 0.07	0.93 ± 0.01
HOG	0.87 ± 0.06	0.83 ± 0.09	0.91 ± 0.05

**Table 2:** Confusion matrix for the maturity stage identification using a color appearance-based NB classifier.

	MS1	MS2	MS3	MS4	*RR* (%)	*OA* (%)
MS1	36	0	0	0	100	99.2
MS2	0	29	0	0	100	
MS3	0	1	28	0	96.6	
MS4	0	0	0	27	100	

**Table 3:** Confusion matrix for the maturity stage identification using a color appearance-based LDA classifier.

	MS1	MS2	MS3	MS4	*RR* (%)	*OA* (%)
MS1	36	0	0	0	100	100
MS2	0	29	0	0	100	
MS3	0	0	29	0	100	
MS4	0	0	0	27	100	

**Table 4:** Confusion matrix for the maturity stage identification using a color appearance-based SVM classifier.

	MS1	MS2	MS3	MS4	*RR* (%)	*OA* (%)
MS1	36	0	0	0	100	99.2
MS2	0	28	1	0	96.6	
MS3	0	0	29	0	100	
MS4	0	0	0	27	100	

**Table 5:** Confusion matrix for the maturity stage identification using the texture feature-based NB classifier.

	MS1	MS2	MS3	MS4	*RR* (%)	*OA* (%)
MS1	35	0	1	0	97.2	92.6
MS2	0	25	4	0	86.2	
MS3	1	3	25	0	86.2	
MS4	0	0	0	27	100	

**Table 6:** Confusion matrix for the maturity stage identification using the texture feature-based LDA classifier.

	MS1	MS2	MS3	MS4	*RR* (%)	*OA* (%)
MS1	34	0	2	0	94.4	86.8
MS2	0	23	6	0	79.3	
MS3	4	4	21	0	72.4	
MS4	0	0	0	27	100	

**Table 7:** Confusion matrix for the maturity stage identification using the texture feature-based SVM classifier.

	MS1	MS2	MS3	MS4	*RR* (%)	*OA* (%)
MS1	35	0	1	0	97.2	93.4
MS2	0	27	2	0	93.1	
MS3	2	3	24	0	82.8	
MS4	0	0	0	27	100	

**Table 8:** Confusion matrix for the maturity stage identification using a local shape feature-based NB classifier.

	MS1	MS2	MS3	MS4	*RR* (%)	*OA* (%)
MS1	31	1	4	0	86.1	84.3
MS2	1	23	3	2	79.3	
MS3	2	4	21	2	72.4	
MS4	0	0	0	27	100	

**Table 9:** Confusion matrix for the maturity stage identification using a local shape feature-based LDA classifier.

	MS1	MS2	MS3	MS4	*RR* (%)	*OA* (%)
MS1	27	0	9	0	75.0	83.5
MS2	1	22	5	1	75.9	
MS3	1	3	25	0	86.2	
MS4	0	0	0	27	100	

**Table 10:** Confusion matrix for the maturity stage identification using a local shape feature-based SVM classifier.

	MS1	MS2	MS3	MS4	*RR* (%)	*OA* (%)
MS1	26	1	9	0	72.2	82.6
MS2	0	24	5	0	82.8	
MS3	3	3	23	0	79.3	
MS4	0	0	0	27	100	

## References

[1] Surya Prabha D., Satheesh Kumar J. (2015). Assessment of banana fruit maturity by image processing technique. J. Food Sci. Technol..

[2] Barragán-Iglesias J., Méndez-Lagunas L.L., Rodríguez-Ramírez J. (2018). Ripeness indexes and physicochemical changes of papaya (Carica papaya L. cv. Maradol) during ripening on-tree. Sci. Hortic..

[3] Hou J.C., Hu Y.H., Hou L.X., Guo K.Q., Takaaki S.S. (2015). Classification of ripening stages of bananas based on support vector machine. Int. J. Agric. Biol. Eng..

[4] Ahmad M.S., Siddiqui M.W. (2015). Factors Affecting Postharvest Quality of Fresh Fruits. Postharvest Quality Assurance of Fruits.

[5] Yossy E.H., Pranata J., Wijaya T., Hermawan H., Budiharto W. (2017). Mango fruit sortation system using neural network and computer vision. Procedia Comput. Sci..

[6] Zhang B.H., Gu B.X., Tian G.Z., Zhou J., Huang J.C., Xiong Y.J. (2018). Challenges and solutions of optical-based nondestructive quality inspection for robotic fruit and vegetable grading systems: A technical review. Trends Food Sci. Technol..

[7] Sofu M.M., Er O., Kayacan M.C., Cetişli B. (2016). Design of an automatic apple sorting system using machine vision. Comput. Electron. Agric..

[8] Mikulic-Petkovsek M., Rescic J., Schmitzer V., Stampar F., Slatnar A., Koron D., Veberica R. (2015). Changes in fruit quality parameters of four Ribes species during ripening. Food Chem..

[9] Sun Y.D., Singh Z., Tokala V.Y., Heather B. (2019). Harvest maturity stage and cold storage period influence lemon fruit quality. Sci. Hortic..

[10] Yan Z., Zheng L.J., Nie J.Y., Li Z.X., Cheng Y. (2018). Evaluation indices of sour flavor for apple fruit and grading standards. J. Integr. Agr..

[11] Prasad K., Jacob S., Siddiqui M.W. (2015). Postharvest Management of Fruits and Vegetables Storage. Lichtfouse E. (eds) Sustainable Agriculture Reviews.

[12] Hoehn E., Gasser F., Guggenbühl B., Künsch U. (2003). Efficacy of instrumental measurements for determination of minimum requirements of firmness, soluble solids, and acidity of several apple varieties in comparison to consumer expectations. Postharvest Biol. Technol..

[13] Doerflinger F.C., Miller W.B., Nock J.F., Watkins C.B. (2015). Variations in zonal fruit starch concentrations of apples—A developmental phenomenon or an indication of ripening?. Hortic. Res..

[14] Mesa K., Serra S., Masia A., Gagliardi F., Bucci D., Musacchi S. (2016). Seasonal trends of starch and soluble carbohydrates in fruits and leaves of ‘Abbé Fétel’ pear trees and their relationship to fruit quality parameters. Sci. Hortic..

[15] Sanaeifar A., Mohtasebi S.S., Ghasemi-Varnamkhasti M., Ahmadi H. (2016). Application of MOS based electronic nose for the prediction of banana quality properties. Measurement.

[16] Khodabakhshian R., Emadi B., Khojastehpour M., Golzarian M.R. (2017). Determining quality and maturity of pomegranates using multispectral imaging. J. Saudi Soc. Agric. Sci..

[17] Li X.L., Wei Y.Z., Xu J., Feng X.P., Wu F.Y., Zhou R.Q., Jin J.J., Xu K.W., Yu X.J., He Y. (2018). SSC and pH for sweet assessment and maturity classification of harvested cherry fruit based on NIR hyperspectral imaging technology. Postharvest Biol. Technol..

[18] Adebayo S.E., Hashim N., Abdan K., Hanafi M., Mollazade K. (2016). Prediction of quality attributes and ripeness classification of bananas using optical properties. Sci. Hortic..

[19] Xie C.Q., Chu B.Q., He Y. (2018). Prediction of banana color and firmness using a novel wavelengths selection method of hyperspectral imaging. Food Chem..

[20] Mohammadi V., Kheiralipour K., Ghasemi-Varnamkhasti M. (2015). Detecting maturity of persimmon fruit based on image processing technique. Sci. Hortic..

[21] Wan P., Toudeshki A., Tan H.Q., Ehsani R. (2018). A methodology for fresh tomato maturity detection using computer vision. Comput. Electron. Agric..

[22] Tan K.Z., Lee W.S., Gan H., Wang S.W. (2018). Recognising blueberry fruit of different maturity using histogram oriented gradients and colour features in outdoor scenes. Biosystems Eng..

[23] Marimuthu S., Mohamed Mansoor Roomi S. (2017). Particle swarm optimized fuzzy model for the classification of banana ripeness. IEEE Sens. J..

[24] Ahmad K.A., Othman M., Mansor A.R., Abu Bakar M.N. (2015). A Fuzzy Learning Algorithm for Harumanis Maturity Classification. Proceedings of the International Conference on Computing, Mathematics and Statistics (iCMS 2015).

[25] Otsu N. (1979). A threshold selection method from gray-level histograms. IEEE Trans. Syst. Man Cybern..

[26] Ojala T., Pietikainen M., Maenpaa T. (2002). Multiresolution gray-scale and rotation invariant texture classification with local binary. IEEE Trans. Pattern Anal. Mach. Intell..

[27] Aglio A., Rios-Sanchez B., Sanchez-Avila C., de Giles M.J. Analysis of local binary patterns and uniform local binary patterns for palm vein biometric recognition. Proceedings of the International Carnahan Conference on Security Technology.

[28] Dalal N., Triggs B. Histograms of oriented gradients for human detection. Proceedings of the IEEE International conference on Computer Vision and Pattern Recognition.

[29] Berrar D. (2019). Bayes’ Theorem and Naive Bayes Classifier. Encycl. Bioinf. Comput. Biol..

[30] Xu Y., Yang J.Y., Jin Z. (2004). A novel method for Fisher discriminant analysis. Pattern Recognit..

[31] Zhang L., Zhou W.D., Chang P.C. (2011). Generalized nonlinear discriminant analysis and its small sample size problems. Neurocomputing.

[32] Karaçalı B., Ramanath R., Snyder W.E. (2004). A comparative analysis of structural risk minimization by support vector machines and nearest neighbor rule. Pattern Recognit. Lett..

[33] Domingos P. (2012). A few useful things to know about machine learning. Commun. ACM..

[34] Martis R.J., Acharya U.R., Min L.C. (2013). ECG beat classification using PCA, LDA, ICA and Discrete Wavelet Transform. Biomed. Signal Process. Control.

[35] Kim H.C., Kim D., Bang S.Y. (2003). Extensions of LDA by PCA mixture model and class-wise features. Pattern Recognit..

